# Cocktail Anti-Tick Vaccines: The Unforeseen Constraints and Approaches toward Enhanced Efficacies

**DOI:** 10.3390/vaccines8030457

**Published:** 2020-08-19

**Authors:** Charles Ndawula, Ala E. Tabor

**Affiliations:** 1 Vaccinology Research program, National Livestock Resources Research Institute, P O. Box 5746, Nakyesasa 256, Uganda; 2 Centre for Animal Science, Queensland Alliance for Agriculture & Food Innovation, The University of Queensland Australia, St Lucia 4072, Queensland, Australia; 3 School of Chemistry & Molecular Biosciences, The University of Queensland, St Lucia 4072, Queensland, Australia

**Keywords:** cocktail tick-vaccines, antigen competition, tick-acaricide resistance, anti-tick vaccines

## Abstract

Ticks are second to mosquitoes as vectors of disease. Ticks affect livestock industries in Asia, Africa and Australia at ~ $1.13 billion USD per annum. For instance, 80% of the global cattle population is at risk of infestation by the *Rhipicephalus microplus* species-complex, which in 2016 was estimated to cause $22–30 billion USD annual losses. Although the management of tick populations mainly relies on the application of acaricides, this raises concerns due to tick resistance and accumulation of chemical residues in milk, meat, and the environment. To counteract acaricide-resistant tick populations, immunological tick control is regarded among the most promising sustainable strategies. Indeed, immense efforts have been devoted toward identifying tick vaccine antigens. Until now, Bm86-based vaccines have been the most effective under field conditions, but they have shown mixed success worldwide. Currently, of the two Bm86 vaccines commercialized in the 1990s (Gavac^TM^ in Cuba and TickGARD*^PLUS^*™ in Australia), only Gavac™ is available. There is thus growing consensus that combining antigens could broaden the protection range and enhance the efficacies of tick vaccines. Yet, the anticipated outcomes have not been achieved under field conditions. Therefore, this review demystifies the potential limitations and proposes ways of sustaining enhanced cocktail tick vaccine efficacy.

## 1. Introduction

Ticks are obligate blood-feeding parasites that are capable of transmitting pathogens both to humans and animals [1,2]. Most of the ticks circulating globally belong to two families—the Ixodidae (hard ticks) and Argasidae (soft ticks); one tick species belongs to the Nuttalliellidae [3]. In the late 1990s, the impact of Ixodid ticks of genera: *Dermacentor*, *Hyalomma*, *Rhipicephalus*, *Haemaphysalis*) on livestock in Africa, Asia and Australia was estimated at ~$USD708m [4], which currently equates to losses of $1.13 billion USD per annum. In 1997, particularly, *Rhipicephalus microplus* species-complex alone was estimated at $13.9–18.7 billion USD per annum globally, which translates to $22–30 billion USD in 2016 [5,6]. Currently, the spread of *R. microplus* species-complex to other regions [7,8,9,10] is likely to increase the burden of tick species infestation and tick-borne diseases globally. On the other hand, Argasid tick species of the genus *Ornithodorus* transmit the African swine fever virus, which causes a fatal haemorrhagic fever disease in pigs that leads to 100% mortality which severely affects the pig-industry of sub-Saharan Africa, Asia, eastern Europe [11]. Despite the fact that great success of Ixodid-tick control has been achieved using acaricides (anti-tick pesticides), there are increasing reports of acaricide resistance [12,13]. Similarly, acaricides could be used to control Argasid ticks [14,15], yet there is still concern over whether acaricides can be applied effectively given the endophilic lifestyle of Argasids [16]. Nonetheless, excessive use of acaricides can lead to accumulation of chemical residues in milk, meat, and the environment [17]. For these reasons, alternative approaches to tick control have been suggested [18], of which vaccination or immunological control is regarded the most promising, environmentally friendly, and sustainable strategy. To date, numerous antigens have been reported to induce protection against Ixodid ticks [18,19,20,21] and less against Argasids [22,23,24]. Earlier, Willadsen [25] questioned whether combining antigens toward enhanced efficacy is a valid hypothesis. In response, research groups have investigated the concept of cocktail vaccines against Ixodid and Argasid ticks, as summarized in Table 1. Until now, however, the concept remains unsubstantiated under field conditions. Therefore, the goal of this review is to examine the probable constraints and approaches for enhancing the efficacy of cocktail tick vaccines. 

### Historical Background of Tick Vaccine Antigens

The concept of tick vaccines was first demonstrated in 1939 [26]. Initially, Trager [26] observed that repeated tick larvae infestations triggered an acquired immune resistance against Ixodid ticks in guinea pig and rabbit models. A similar phenomenon was observed when guinea pigs were inoculated with native protein tissue-extracts from *Dermacentor variabilis* ticks [27]. Subsequently, in 1940, Trager [28] demonstrated that Argasid ticks can also induce partial acquired immunity in chickens. The acquired immune resistance was determined to be based on a reduced number of engorged ticks, reduced blood-feeding, and reduced weight and viability of eggs [29]. Then, the question arose as to how the acquired immune resistance affects the ticks’ physiological parameters. Partially in response, different research groups have reported that the hosts’ antibodies/immunoglobulins (IgGs) can traverse the tick gut epithelium to the hemolymph and other tick tissues [30,31,32]. Furthermore, the antibodies induced against particular tick vaccine antigens are shown to react against the corresponding tick tissue proteins [33]. Therefore, it is presumed that when ingested during blood feeding, the anti-tick antigen sera could interfere with the physiological functionality of internal tick proteins. 

Building on these observations, numerous recombinant tick antigens have been identified against Ixodid ticks [18,19,20,21], of which Bm86 is still the most successful under field conditions [34,35]. Additionally, Bm86 is shown to induce cross-protection against different tick species, but not against species such as *Amblyomma variegatum* and *Rhipicephalus appendiculatus* which affect livestock in Africa [36]. Other tick vaccine antigens—for instance, subolesin [37], glutathione S-transferase (GST) [38,39], cement protein (64TRP) [40]—have also been reported to induce cross-protection against different Ixodid ticks. By contrast, although Trager [28] and several other research groups [22] have demonstrated that Agarsid tick-extracts can induce acquired immunity *in vivo*, the progress toward the development of subunit vaccines against Argasids has been slow. Certainly, there are reasons for this slow progress which will not be addressed in this review. Therefore, it is not coincidental that to date only a few single vaccines against Argasids have been reported [22,23,24]. Nonetheless, tick researchers still aim to develop good vaccines which are able to induce a substantial humoral/or cell mediated protective immune responses in Argasid and Ixodid ticks. However, that is not the only factor that determines an ideal anti-tick vaccine. For instance, with reference to Ixodid ticks, Díaz-Martín et al. [22] presented the desired attributes of an ideal anti-tick vaccine. In general, the efficacy of a particular tick-vaccine can be influenced by factors such as the immunogenicity of the proteins and the host’s immune response. 

Finally, in comparison to other vectors and pathogens, ticks are reported to have very large genomes (2.1–7.1Gb) [41,42]. Moreover, tick genomes are highly repetitive—a phenomenon that could be fundamental to tick survival. It is, therefore, likely that ticks express a plethora of proteins depending on their environment. This further supports the hypothesis that the provision of cocktail vaccine antigens could enhance anti-tick protection efficacy [25]. 

## 2. Approaches to Identifying Cocktail Vaccine Antigens

### 2.1. Single-Antigen Vaccine Efficacy 

Often, a cocktail tick vaccine is constituted with antigens that were previously identified based on their individual protection efficacy. Presumably, the rationale is that if antigens A and B induce protection of 45% and 55%, respectively, combining the two antigens could double the protection efficacy. Interestingly, a similar approach has been embraced toward enhancing the efficacy of vector-borne pathogen vaccines [43,44]. Even though the approach is direct, simple, and mathematically logical, it does not take into account the potential immunological shortcomings of combining antigens. These will be discussed herein later.

Alternatively, (as shown in Table 1) several research groups have tested cocktail vaccines without previously determining individual antigen efficacies. This could be due to high cost of livestock for trial experiments. However, in this case, the cause for combining two or more antigens (to enhance the efficacy of one antigen) cannot be justified. Of course, blindly formulated cocktail vaccines may induce numerous effects, especially if the antigens were derived from proteins which play different roles in tick physiology. Nevertheless, immunological shortcomings due to combining antigens are inevitable. 

### 2.2. Antigen Serum Immuno-Cross-Reactivity

Recently, the principle of independent immunogenicity of tick vaccine antigens has further been investigated toward selecting cocktail antigens [45]. Specifically, Ndawula et al. [45] demonstrated that serum independently induced against the recombinant glutathione S-transferase (rGST) cross-reacts against heterologous tick species rGSTs, although at varying intensities. Interestingly, a similar approach was earlier used toward identifying a potential broad-spectrum single tick vaccine antigen based on tick cement [40]. The approach of serum cross-reactivity, however, can only be applied while selecting among homologous tick antigens.

### 2.3. Antigen Discovery Approaches

Single tick vaccine antigens are often developed based on the genetic make-up of the tick species of interest. Indeed, the approach could also be used for identifying cocktail tick vaccine antigens. Historically, methods used commonly included RNA interference [46], expression library immunization (ELI), evaluation of expressed sequence tags [47], interactomics [48], proteomics [47], and transcriptomics [48]. However, it should be noted, that even though RNAi can be used for identifying tick antigens [49], the method is designed for studying the potential roles of target genes in tick physiology and as such may not be immunogenic [50,51]. Of the described methods, transcriptomics is most commonly used and further has been used in combination with proteomics [52,53], and metabolomics [54]. This approach has enhanced the efficiency and accuracy of antigen discovery, which could also hasten the identification of potential cocktail antigens.

### 2.4. Antigen-Serum-Induced Effect

In the quest for methods to control ticks and tick-borne diseases, feeding of ticks in the laboratory remains a major challenge; thus, the use of laboratory animals is preferred. However, animals are expensive, and their use raises ethical debates. Therefore, *in vitro* or artificial tick-feeding methods have been developed with the establishment of capillary [55,56], glass tube [57], and membrane [58,59], tick feeding protocols. 

Artificial membrane tick feeding has since been used, for instance, in studying the effect of novel acaricide molecules on tick physiology [60], and proteins involved in pathogen transmission as targets for vaccine development [61]. Additionally, although artificial membrane feeding has been successfully used for growing ticks *in vitro*, the method requires twice daily defibrinated blood changes, and it can take up to eight weeks for all stages to emerge [62]. The method has been commonly used for acaricide screening but is less amenable for high throughput screening of vaccine candidates. By contrast, the capillary feeding method is easier, so it is commonly used to feed semi-engorged female ticks [63,64,65]. The limitation to this method is that the capillaries become blocked by blood hence capillaries are changed regularly. To prevent tube blockage, blood must be preserved in anti-coagulants during collection [53,59]. A concern has arisen as to whether different anti-coagulants can affect tick physiology and development. Thus, Lew-Tabor et al. [53] used glass tubes to feed semi-engorged adult female *R. australis* with antibodies or blood preserved with different anticoagulants and assessed the effect induced on the tick-weight, egg-weight and egg-viability. The findings showed that 1mg/ml of Bm86 IgG induces the same efficacy (*in vitro*) as Bm86 vaccine (*in vivo*), which suggests that the model is reliable for screening candidate tick-vaccines.

Recently, Trentelman et al. [66] exploited *in vitro* tick feeding for larval stages to examine the effect of a cocktail anti-tick vaccine serum. They demonstrated that a combination of anti-Bm86 and anti-subolesin serum inhibited the feeding of *Rhipicephalus australis* larvae, which suggested that the antigens were candidates for a cocktail vaccine. On the contrary, however, Perner et al. [67] showed that feeding adult ticks on a meal devoid of haemoglobin leads to egg-sterility. This raises a question whether the feeding-inhibition effect induced on *Rhipicephalus australis* larvae [66] was partly due to the lack of haemoglobin. Following feeding, larvae were weighed, but moulting to the nymph stage was not examined. In addition, although a blood anti-coagulant was used in the study to ensure the ticks are able to feed continuously on the blood provided, there is a possibility that anticoagulants affect tick development. Along these lines, Lew-Tabor et al [53] demonstrated that eggs laid by ticks fed on heparinized blood were viable, but not viable when ticks were fed with EDTA or CPDA blood. Therefore, while conducting *in vitro* tick feeding, the experiments are not deemed successful unless the next tick-life stage emerges.

Even though this discussion is based on reports from Ixodid artificial feeding experiments, we speculate that the same principles could apply to artificial feeding studies for Argasid (soft) ticks. Our hypothesis is based on evidence that *in vitro* artificial feeding membranes have been exploited for the maintenance of Argasid ticks (*O. coriaceus* and *O. moubata*) [68,69,70] and in acaricide-efficacy studies [14,15]. However, reports that examine the effect of cocktail vaccines against Argasids using in vitro artificial feeding membranes are yet to emerge. Nevertheless, artificial feeding is an important tool not only in search of vaccines against Ixodids (hard ticks) and Argasids (soft ticks), especially in the selection of tick antigens for cocktail tick vaccines. For instance, the model could be used to determine the concentration of cocktail vaccines. This view is based on evidence that vaccine protection efficacy is directly proportional to vaccine-induced antibodies [71]. The tool could also be used to assess the vaccine effect on different stages of tick development. 

## 3. Potential Constraints toward Cocktail Vaccine Efficacy

### 3.1. Antigenic Competition

To date, numerous cocktail vaccines against Ixodid ticks and a few against Argasid ticks have been investigated (Table 1) with the hypothesis that combining at least two antigens could increase the protective antibody response. However, often no enhanced protection is observed coupled with reduction of antibodies against each of the cocktail antigens [72,73,74]. In 1902, a German immunologist, Michaelis, made a similar observation [75]. Specifically, Michaelis noted that following the inoculation of a cocktail of antigens, the immune response to one antigen was suppressed by the response to a second, unrelated antigen. This phenomenon is described as antigenic competition. Indeed, this phenomenon sparked interest among immunologists—to date, over 10 mechanisms have been proposed to explain the reduction of antibodies in cocktail vaccines. Pross and Edinger [76] thoroughly discussed the research advances that were made toward understanding the mechanism of antigenic competition. This phenomenon could also account for the reduction of immune responses against cocktail tick vaccines, but the mechanism of action remains a topic of research. Furthermore, Taussig et al. [77] revealed that the reduction of antibodies can result from inter or intra-molecular competition. Intramolecular and intermolecular competition occurs between the determinants on the same or different immunogen(s). It is therefore probable that intermolecular competition is common within cocktail tick vaccines consisting of heterologous or homologous antigens. Thus, the fundamental question remains; could the reduction of antibodies against the antigens explain why cocktail tick vaccines are yet to show enhanced efficacy under field conditions? It seems likely that antigenic competition is further influenced by other factors.

#### 3.1.1. Antigen Concentration

Similar to other vaccines, the efficacy of tick vaccines is determined based on humoral immune responses [78]. Indeed, antigen concentration is one of the factors that influences humoral immune responses. Given that cocktail tick vaccines are aimed at enhancing efficacy, it is tempting to use a higher concentration of single antigens. Suppose that 100 µg of vaccine A and B independently induced 45% and 55% efficacy, 100 µg of A and B may be combined to make a cocktail vaccine. Indeed, this approach is mathematically logical, but it may trigger undesired immune responses which include

1. Increased antigenic competition. For instance, evidence indicates that inter-molecular competition increases with dose concentration [77,79,80].
2. Immunotolerance. This is a condition where the immune system shows a reduced response against an antigenic substance or molecule due to prior exposure. The response is classified into high and low immunotolerance that is triggered by high or low-dose antigen concentrations, respectively [81,82]. Furthermore, the tolerance can be influenced by other factors such as the route of immunization, antigen protein molecular weight, and immunogenicity [83]. Overall, the tolerance induction mechanism is based on whether the antigens are T-cell dependent or independent [84,85]. However, to date, the optimum concentration for formulating cocktail tick vaccine antigens is still unknown and is likely to vary depending on the antigens within the cocktail.

#### 3.1.2. Antigen–Adjuvant Interaction

The development of anti-tick vaccine antigens (subunit vaccines) mainly relies on the advances in recombinant DNA technology. In contrast to other forms of vaccines- based on native proteins, live or attenuated microorganisms- subunit vaccines can be manufactured, purified and as such, are safer. However, considering that subunit vaccines are composed of recombinant proteins, vaccines are also prone to degradation. The main drawback to subunit vaccines is that they may not elicit a sufficient protective immune response or can be poorly immunogenic [86]. Therefore, adjuvants are used to avert the limitations of subunit vaccines [87,88]. It should be noted that adjuvants act either as immunopotentiators or delivery systems [89,90]. For most anti-tick vaccine research to date, adjuvants have been involved as the primary delivery system. For instance, a lower tick rejection was reported against tick salivary gland antigen extract with Freund’s complete adjuvant (FCA) compared to Freund‘s incomplete adjuvant (FIA) [91]. Often pilot antigen *in vivo* studies use a combination of FCA followed by FIA boosts, see Table 1 [72,92,93]. Imamura and co-workers [92] for instance, demonstrated that *Rhipicephalus appendiculatus* recombinant serine protease inhibitors (rRAS-1 and 2) delivered as a cocktail vaccine induced higher tick mortality with an adjuvant combination (FCA and FIA) compared with the single adjuvant (FIA). Even though the efficacies were not high enough to warrant commercialization of the cocktail vaccine (rRAS-1 and 2), the study demonstrated the variable impact of different adjuvant combinations. However, currently Freunds’ adjuvants are not recommended for commercial use in large animals, as they induce tissue damage and painful reactions following vaccination [94]. For these reasons, alternatives to Freunds’ adjuvants, the Montanide (‘oil-in-water’) adjuvants have been used in several cocktail studies summarized in Table 1 [38,45,74,95,96,97,98]. Until now, however, few studies have examined the effect of different adjuvants on cocktail vaccine efficacies [73,92,95,99]. Nonetheless, it is impossible to rule out the possibility that adjuvants impact the efficacy of cocktail tick vaccine antigens.

#### 3.1.3. Animal Genetics

Presumably, because of logistical constraints, tick vaccination experiments are conducted using animal models such as rabbits, mice, guinea pigs, sheep, and dogs. Often the criteria for selecting the models are (1) whether the ticks can feed on the animal model, (2) whether the animal has had prior tick exposure (discussed below), and (3) the availability of pathogen-free ticks for challenge. Another, but rarely scrutinized, factor is the animal’s genetic background. Reports indicate that genetic factors can influence the animal immune response [100,101,102,103]. Moreover, the influence is shown to be high among inbred models [104,105,106,107]. Furthermore, Taussig et al. [108] demonstrated that antigenic competition varies with the genetic background of the experimental animal. Intriguingly, a similar phenomenon was observed among rabbits that were inoculated with the same recombinant glutathione S-transferase (rGST) cocktail tick antigen [109]. In this study, two rabbits of each group were inoculated as follows: group 1, cocktail 1 made of rGSTs from *Rhipicephalus decoloratus* (rGST-Rd), *Amblyomma variegatum* (rGST-Av) [45] *Haemaphysalis longicornis* (rGST-Hl) [110], and group 2, with cocktail 2 made of rGST-Av and rGST-Rd. Note, however, in that report, the genetic background of the rabbits was not known. Based on this study, it is possible that the potential of the cocktail tick vaccine antigens could be underestimated; hence, the vaccines are not further investigated. 

Additionally, the question of whether prior animal tick exposure (which can lead to bovine resistance to ticks) may influence the efficacy of candidate cocktail vaccines. Although all tick species induce cattle resistance, the phenomenon is more pronounced when cattle are exposed to ‘one-host’ tick species such as *R. decoloratus* and/or *R. microplus.* In fact, vaccination studies against ‘one-host’ ticks can only be conducted using cattle. Specifically, these ticks complete their entire life cycle on the same cow or bull. One-host tick species evolved on *Bos indicus* breeds of cattle which, as such, are capable of tolerating ticks at lower numbers compared to tick susceptible *Bos taurus* breeds [110]. For this reason, in tropical and sub-tropical regions of the world, *Bos indicus* x *Bos taurus* breeds are used to control tick populations. Consequently, these cross breeds continue to show traits of widespread tick resistance [111]. These findings emphasize the urgent need for research to identify genetic markers to select cattle for tick resistance particularly in crossbreeds reviewed by Tabor et al. [112]. In addition, often cattle tick vaccine trials are undertaken in susceptible *Bos taurus* breeds, it is rare that trials are undertaken using several different breeds or crossbreeds. It is possible that variable responses to vaccine candidates will also be observed in cattle, such as a recent report using subolesin antigens in *Bos indicus* vs. a crossbreed [113] (see Table 1). 

Interestingly, Piper et al. [111] indicated that ELISA screening using tick fractions showed low IgG responses from serum from tick exposed *R. microplus* resistant cattle. This has also been observed in our tick vaccine research, where different proteins induced high IgG responses in susceptible cattle breeds, but these did not control ticks following infestation challenge [72]. 

Finally, considering that cattle trials are very expensive, in some instances, antibody responses are first measured in sheep as a cheaper model than cattle [114]. The results, however, do not always translate to how the antigens will behave in cattle as confirmed by Bm86 cocktail vaccination studies in sheep and cattle [73]. Given these potential limitations, it is worth taking into account the major histocompatibility complex (MHC) gene diversity among different cattle breeds when considering cocktail vaccines. MHCs are groups of genes that code for proteins found on the surfaces of cells that help the immune system recognize foreign substances. There are two types of genes coding for the proteins—MHC class I molecules and MHC class II molecules that are directly involved in the antigen presentation. These genes are highly polymorphic and are less defined in species such as cattle. Several tick immunological studies have demonstrated that different MHC2 classes are responsible for tick resistance or susceptibility [115,116]; however, these findings have not been used to inform vaccine research directly. Some researchers have tried to predict B cell and/or T cell binding epitopes in putative tick vaccine candidates (reviewed by Lew-Tabor and Rodriguez Valle [6,117]); however, the tools for these bioinformatics predictions for use in non-human hosts have yet to be developed. It is likely that these factors could still be hindering the success of researchers in formulating cocktail tick vaccine with high immune potency.

### 3.2. Subunit Protein Expression System

Since the inception of tick vaccines against Ixodid [26] and Argasid [28] ticks, numerous antigens have been identified [18,19,20,21,22]. and the respective proteins (tick antigens) have been expressed in different systems. The commonly used systems include mammalian, yeast, bacterial-based, and insect cells [118,119,120], all investigated toward obtaining immunogenic vaccines (see also Table 1). The rationale is that the conformational structure influences vaccine immunogenicity. Particularly, expression of proteins in a bacterial system could lead to the formation of misfolded proteins which lack the conformational epitopes that induce antibody production [121]. On the contrary, although Bm86 protein expressed in *E. coli* is less immunogenic than the Bm86 expressed in yeast [18] or insect [95] cells, no significant reduction in vaccine efficacy is reported [122]. The *E. coli* expressed Bm86 ‘glycoprotein’ lacked glycosylation compared to yeast expressed Bm86 [123,124]. Although bacterial expression presents numerous advantages over other systems [125], it should be noted that bacterial-expressed proteins are not likely to have the same biological activity as the corresponding native eukaryotic tick protein. Currently, it is evident that the bacterial-based systems (particularly *E. coli* cells) is the preferred system for expressing subunit tick proteins (Table 1). However, this choice may simply be the preferential use of bacteria in these studies. In addition, until now there is no benchmark system for expressing anti-tick vaccines [126], with ‘anecdotal evidence’ indicating that eukaryotic expression systems are better than prokaryotic.

Considering that the concept of cocktail vaccines calls for the application of at least two single or chimeric proteins antigens, there is a possibility that conformational structure-alteration of *E. coli*–expressed cocktail-antigens affects the immunogenicity of cocktail vaccines. Although cocktail vaccines based on single antigens have been tested (Table 1), reports of chimeric-based vaccines are scarce. The scarcity of reports on chimeric-based cocktail vaccines is not an indicator that tick-researchers have not made chimeras; rather, it could suggest that tick researchers are still facing challenges in expressing effective chimeric-tick vaccines. Hypothetically, introducing large genetic material (chimera) into *E. coli* (the most commonly used system for expressing tick proteins) still limits the expression of chimeric-tick vaccines. This view is based on evidence that methods (for instance, electroporation) for delivering genetic material can lead to, cell lysis, high cell mortality, low transformation efficiency, and low throughput [127,128,129]. Another widely used method for transformation is heat shock which can only deliver small sized plasmids [130]. A study comparing the effectiveness of different expression platforms to deliver chimeric tick vaccines would be beneficial. However, this potentially brings large costs to researchers associated with large animal trials and also explains why perhaps such studies have yet to be conducted or published. 

## 4. Can We Enhance the Efficacy of Cocktail Vaccines?

In spite of the aforementioned potential limitations, this section discusses the probable approaches of enhancing cocktail tick vaccine antigens.

### 4.1. Cocktail Antigen Selection

In principle cocktail tick vaccines are formulated with the goals of (A) enhancing the protection efficacy against a particular tick species, (B) increasing the tick species host protection range, (C) inducing protection against different stages of tick development, or (D) interfering with tick-borne pathogen transmission and the tick biological parameters. Therefore, tick-borne pathogen antigens, antigens from different tick stages, and from different tick species could be used to formulate cocktail vaccines [6]. However, antigenic competition is likely to occur between the cocktail antigens. Then, the question remains: which antigens should be used to formulate cocktail vaccines? For instance, recently, it was illustrated a probable approach for selecting cocktail rGST antigens (Table 1) [45]. It should be noted that the antigens were selected based on the anti-rGST serum cross-reactivity rGSTs from five different tick species. Subsequently, cocktail 1 (*R. decoloratus* rGST-Rd, *A. variegatum* rGST-Av and *H. longicornis* rGST-Hl) and cocktail 2 (rGST-Rd and rGST-Av) were combined and investigated in rabbits against *Rhipicephalus sanguineus* [45], and *R. appendiculatus* infestation [109]. Previously, Parizi et al. [96] investigated a cocktail vaccine that was composed of rGST-Hl from *H. longicornis* [110], vitellin-degrading cysteine endopeptidase (VTDCE) [131], and *Boophilus* yolk pro-cathepsin (BYC) [132] from *R. microplus*. Although the cocktail vaccination experiments were performed in different models (rabbits and cattle respectively), a reduction of antibodies against the cocktail antigens was noted in both studies. By contrast, reduction among the related rGST cocktail of related antigens [45,109] was less than that shown with the cocktail of non-related antigens [96]. However, no direct comparison could be made regarding the efficacy of the rGST cocktails [45,96,109] as the experiments were conducted against different tick species. Nevertheless, the impact induced on the biological parameters of *R. appendiculatus* [109] was substantively significant compared to *R. sanguineus* [45]. 

Along these lines, Hammerl et al. [133] reported that heterologous anti-cocktail serum (induced against related but not identical antigens) consisted of diverse antibodies that could cross-react against both the cocktail antigens and other weak immunogens. In fact, antiserum cross-reactivity is suggested to be fundamental in inducing heterologous adaptive immunity, a phenomenon where a pathogen vaccine antigen induces immune-protection against non-related pathogens [134]. For instance, the implication of heterologous immunity in the control of human infectious pathogens has been extensively discussed [135,136,137]. Therefore, it is likely that heterologous cocktail tick vaccine (with homologous, but not identical antigens) could induce lower antigenic competition and a higher cross-reactive adaptive immunity than the cocktails made-up of non-related, or non-identical antigens. Additionally, the cross-reactive adaptive immunity could be further exploited in the search for broad-spectrum cocktail tick-vaccine antigens.

### 4.2. Chimera-Based Cocktail Tick Vaccines

Chimeras are structural-based hybrid vaccines that are composed by fusing two or more antigenic fragments. In this context, antigen nucleotide coding sequences are fused using a linker sequence and inserted into an expression plasmid. This approach has been investigated for the control of ticks and tick-borne pathogens [138,139,140]. It should be noted that the chimera vaccines were constituted based on the tick antigen open reading frame (ORF) and a pathogen protein-coding sequence. Until now however, the reports on chimera vaccines based on several tick antigen nucleotide coding sequences remain scarce with no approaches leading to commercialization. Nonetheless, it is feasible that chimeric tick sequence constructs can be expressed as subunit/ recombinant vaccine antigens. Indeed, to limit the logistical constraints, expression can be performed in *E. coli* or yeast. However, as predicted with subunit vaccines the expressed protein is likely to be misfolded and less immunogenic [121]. These limitations could be addressed in two ways discussed hereafter. However, for reasons described (3.2), it is probable that in both approaches researchers are likely to encounter challenges in delivering large chimeric plasmids in *E. coli* (the commonly used system for tick protein expression) which will hinder success in chimeric-cocktail antigen expression. To avert this challenge researchers ought to consider using the cell penetration peptide mediated transformation method for inserting chimeric plasmids [141].

**Hypothesis** **1.** *Construction of epitope-based chimera vaccines.*

Evidence indicates that epitope-based synthetic peptide vaccines can independently, induce immune protection against ticks [72,142,143]. Consequently, the possibility of using epitope-based cocktail vaccines has been demonstrated [60], although there was no enhanced vaccine efficacy (Table 1). Rather, on combining the epitope-synthetic peptides, there was a decrease in the humoral response which could have affected the overall efficacy. Certainly, the potential causes earlier discussed herein could apply to this phenomenon. For instance, evidence shows that an epitope-based cocktail may also exhibit immunological limitations such as antigenic competition [144,145]. Nevertheless, the factors proposed hereafter toward enhancing the efficacy of cocktail tick vaccines could suffice. Despite the fact that synthetic-peptide-based vaccines present numerous benefits, these vaccines are also associated with high production costs which limits their applications within livestock industries [146]. Therefore, designing epitope-based chimeric vaccines is one of the ways of limiting the production costs. Although the concept of epitope-chimera vaccines has been exploited toward the control of ticks [147], reports of enhanced protection under field conditions remain scarce. This could be attributed to the fact that there are still no standard pipelines for predicting tick vaccine epitopes [148,149,150].

**Hypothesis** **2.** *Chimera-based DNA vaccines.*

Chimeric constructs could be delivered as DNA vaccines. Indeed, the merits and demerits of DNA vaccines have been discussed extensively [151]. The concept of DNA tick-vaccines has been demonstrated [152,153]. Similarly, chimeric DNA constructs could be combined to formulate cocktail vaccines, however, this could also trigger undesired immunological reactions such as antigenic competition [154]. Therefore, to ensure efficacy of chimera-based vaccines, the factors discussed hereafter should be taken into account.

### 4.3. Conjugate Vaccines

Based on the evidence that native protein extracts induce an acquired resistance against ticks [26], various tick recombinant antigens have been identified [18,19,20,21]. With the exception of Bm86 [34,35], few antigens have shown potential to be applied under field conditions. Generally, subunit antigens confer a lower immune response compared to the native protein extracts. This could be attributed to the fact that, unlike subunit vaccine proteins, native proteins contain post-transcriptional modifications such as glycosylation [155,156]. In contrast, it seems that some subunit antigens contain glycosylated and/or non-glycosylated determinants. For instance, the Bm86 protein is a glycosylated antigen that was isolated from *R. microplus* [157]. In addition, the antigen was shown to induce protection against *R. microplus* [95] and other tick species [36]. Intriguingly, the Bm86 protein expressed in *E. coli* was shown to induce a lower immune response than the protein expressed in insect cells [95] and *Pichia pastoris* [47]. This low immunogenicity could be attributed to the fact that the proteins expressed in *E. coli* are not glycosylated [158]. In fact, Willadsen and McKenna [122], illustrated that antibodies induced against the Bm86 protein expressed in *E. coli* could not react against the native gut protein extract. 

Evidence shows that when used independently, polysaccharide antigenic determinants induce low immunogenicity [159,160]. Furthermore, it was illustrated that when a polysaccharide is conjugated to a protein carrier, its immunogenicity is enhanced [160]. This concept has since been used to enhance the protective efficacy against infectious human pathogens [161,162]. The probable benefit is that the conjugate vaccine triggers a dual immune response. Specifically, the polysaccharide and protein carrier induce a B cell and T cell immune responses respectively which results in increased B cell activation and hence antibody production [163,164]. Given that glycoproteins are key in the tick-pathogen interaction [165,166], and in the induction of host acquired immune tick resistance [18,157], a cocktail of glycosylated and non-glycosylated tick-antigens could enhance vaccine protection efficacy.

### 4.4. Modification of the Cocktail Vaccination Protocols

Similar to traditional vaccines, anti-tick vaccination is commonly based on the principle that the animal immune system is primed with the tick antigen and subsequently boosted with the same antigen. Specifically, this is referred to as the homologous prime-boost immunization strategy [167]. Despite the fact that this immunization strategy is also used with cocktail tick vaccines, there are no reports on whether the approach potentiates the antigens. It is also not known whether the interval between the cocktail vaccine doses is sufficient to limit the interference of the boost dose-response with the primary immune germinal cells. For instance, in humans, single vaccine doses are administered at an interval of four or eight weeks [168]. By contrast, tick vaccine antigens are often administered at intervals of 2–3 weeks. Given that the anticipated potential of the cocktail antigens is yet to be fully exploited using the homologous prime-boost vaccination, it is worth investigating the heterologous prime-boost strategy. However, vaccination using the latter approach may require different delivery systems [169]. For instance, the heterologous strategy may involve priming with the host with a DNA-based antigen and boosting with a subunit or peptide antigen. In fact, recently, Hassan et al. [153] demonstrated the potential of enhancing a tick vaccine using the DNA prime dose and a synthetic peptide. 

While undertaking cocktail vaccine antigen experiments, it is worth asking the following questions: (A) what is the best immunization strategy? (B) what is the prime boosting antigen? (C) how many boost doses should be administered? (D) what is the best site for cocktail vaccine inoculation? Evidence, for instance, indicates that inoculation of multiple antigens at the same site induces antigenic competition [80,170]. (E) could adjuvants play a role in the cocktail vaccine antigen efficacy? For example, Brown et al. [91] demonstrated that a salivary gland protein in FIA was more immunogenic than in FCA; however, this is contradicted by the reports of Imamura et al. using different salivary protein antigens (Table 1). 

## 5. Conclusions

The potential limitations and probable ways discussed herein toward the enhanced efficacy of cocktail tick vaccines are by no means the only factors. For instance, alternative vaccine delivery systems (VDSs) such as immune-stimulating complexes (ISCOMs), liposomes, and nanoparticles [90] are yet to be exploited not just with the formulation of cocktail anti-tick vaccines but with single anti-tick vaccines as well. Effective vaccine delivery systems are a necessity, especially when antigens are rapidly degraded during inoculation and hence not efficiently transported or presented to the immune system. Nevertheless, the factors discussed in this review merit experimental studies, not just in validating the previous cocktail vaccines but also in formulating novel cocktail vaccines against Ixodid and Argasid ticks. Calculating the protection efficacy of cocktail vaccines may require initial screening of the independent single antigens. Based on the evidence illustrated herein, it is no longer a question as earlier raised by Willadsen [25] that the concept of cocktail antigens is a valid hypothesis; the hope of sustaining enhanced efficacy has been substantiated [97]. Additionally, using an in vitro tick feeding with cocktail vaccine antibodies, Trentelman et al. [66] demonstrated that anti-Bm86 antibodies attacked the tick gut, whereas anti-subolesin antibodies attacked tick salivary glands and the rectal sac epithelium. This illustrates potential synergistic benefits of using cocktail tick vaccines. Ultimately, there is cause for optimism that cocktail antigens can boost the effort toward controlling ticks under field conditions. 

## Figures and Tables

**Table 1:** Summary of single and cocktail efficacies from *in vivo* tick vaccine trials.

Cocktail Constituting Antigens	Subunit Protein Expression System	Cocktail Vaccination Schedule	Target Tick Species or Pathogen	Single Antigen Vaccine Efficacy (E) *	Cocktail Antigen Vaccine Efficacy (E) *	References
*Rhipicephalus microplus*-gut glycoprotein (Bm86) and *R. microplus*-putative carboxydipeptidase (Bm91)	Bacterial (*Escherichia coli*)	**Dose: 100** μg per antigen	*Rhipicephalus* (*Boophilus*) *microplus*	Bm86: 80%	Overall dual efficacy data not reported.	[95,171,172]
		**Adjuvant:** Montanide 888 (Seppic) and Marcol 52.				
		**Intervals:** not indicated (two doses)		Bm91: 37% * Reduction in egg weights relative to controls	* Number of engorged ticks, egg weight and Tick weight/egg weight	
		**Model:** Cattle				
*Rhipicephalus microplus*-mucine-like glycoprotein (BMA7) and *R. microplus*-gut glycoprotein (Bm86)	Protein chromatography	**Dose: 219** μg per antigen	*Rhipicephalus* (*Boophilus*) *microplus*	BMA7: * Reduction in egg weights relative to controls.	Overall dual efficacy data not reported.	[74,95]
		**Adjuvant:** Montanide ISA 70 V.				
		**Intervals:** four weeks (two doses)		(overall efficacy data not reported)	* Reduction in egg weights relative to controls.	
		**Model:** Cattle		Bm86: 80%		
*Ixodes scapularis-*putative protein: 4E6, Nucleotidase-like (4F8 and Subolesin (4D8)	Bacterial (*Escherichia coli*)	**Dose:** 50μg per antigen	*Ixodes scapularis*	4E6: 40 ± 38%	58 ± 11% * Number of engorged ticks and oviposition	[114]
		**Adjuvant:** FIA (Freund’s complete adjuvant)		4F8: 33 ± 9%		
		**Intervals:** 0, 6, 12, 14 weeks		4D8: 71 ± 36% * Number of ticks and oviposition		
		**Model:** Sheep				
*Ixodes scapularis-*putative proteins: Nucleotidase-like (4F8) and Subolesin (4D8)	Bacterial (*Escherichia coli*)	Dose: 50μg per antigen	*Ixodes scapularis*	Larvae	Not done	[138]
		Adjuvant: FIA (Freund’s complete adjuvant)		4F8: 62%, 4D8: 71%, 4E6: 63% * Number of replete larvae and moulting-inhibition		
or *I. scapularis-*putative proteins synthetic peptide (4E6)		Intervals: 0, 4, 7 weeks				
		Model: Rabbits and mice	*scapularis*			
				**Nymphs**	**Nymphs**	
				4D8: 35%, 0%	63%, 0% * Inhibition of nymph infestation and weight of engorged nymph	
				4F8: 39%, 0%		
				4E6: 0%, 0% * Inhibition of nymph infestation and weight of engorged nymph		
				**Nymphs**	**Nymphs**	
			*Dermacentor variabilis*	4D8: 22%, 32%	8%, 0% * Inhibition of nymph infestation and weight of engorged nymphs	
				4F8: 0%, 0 %		
				4E6: 5%, 27% * Inhibition of nymph infestation and weight of engorged nymphs		
				**Nymphs**	**Nymphs**	
			*Amblyomma americanum*	4D8: 17%, 3%	12%, 16%	
				4F8: 9%, 1%	* Inhibition of nymph infestation and weight of engorged nymphs	
				4E6: 29%, 0% * Inhibition of nymph infestation and weight of engorged nymphs		
*Rhipicephalus appendiculatus* serpin-1 (rRAS-1), serpin-2 (rRAS-2)	Bacterial (*Escherichia coli*)	**Dose:** 500 μg per antigen	*Rhipicephalus appendiculatus*	(Not done)	61.4% * Reduction in nymph engorgement and 28 (male) and 43% (female) * increased tick mortality	[99]
		**Adjuvant:** Freund’s complete adjuvant (FIA) (Priming dose), and Freund’s incomplete adjuvant (FAC) (booster dose)				
		**Intervals:** 14 days (three doses)				
		**Model:** Cattle				
*Rhipicephalus appendiculatus-*protein: serpin-3 (rRAS-3), serpin-4 (rRAS-4) and a 36kDa immuno-dominant protein (rRIM36)	Bacterial (*Escherichia coli*)	**Dose:** 300–350 μg per antigen	*Rhipicephalus appendiculatus*	Note done	39.5% * uninfected-tick mortality	[92]
		**Adjuvants:** Freund’s complete adjuvant (FCA) (Priming dose) and Freundś incomplete adjuvant (FIA) (booster doses)	*Theileria parva*		48.5% * *T. parva* infected-tick mortality	
		**Intervals:** 14 days (three doses)				
		**Model:** Cattle				
*Rhipicephalus microplus-*5´-nucleotidase (4F8) and *R. microplus*-gut glycoprotein (Bm86)	4F8: Bacterial (*Escherichia coli*)	**Dose:** 80 and 100 μg per antigen in the respective models	*Rhipicephalus microplus*	4F8: Overall efficacy data not reported	Overall efficacy data not reported.	[73]
	Bm86 (Yeast: *Pichia pastoris*)	**Adjuvant:** ISA50, QuilA and ISA773 (Seppic)		Bm86: 81%		
		**Model:** Sheep and cattle		* Number of engorged ticks, egg weight and tick weight/egg weight	* Number of engorged ticks, egg weight and tick weight/egg weight	
*Haemaphysalis longicornis*-Recombinant Glutathione-S. transferase (rGST-Hl), *Rhipicephalus microplus-*vitellin-degrading cysteine endopeptidase (VTDCE) and *R. microplus*-*Boophilus* Yolk Cathepsin (BYC)	Bacterial (*Escherichia coli*)	**Dose:** 200 μg per antigen	*Rhipicephalus microplus*	rGST-Hl: 57%	51.3–61.6%	[38,96,173,174]
		**Adjuvant:** Montanide 888 and Marcol 52		VTDCE: 21%	* Number of engorged ticks	
		**Intervals:** three weeks (three doses)		BYC: 25.24% * Number of of engorged ticks, egg weight and larva-emergence		
		**Model:** Cattle				
*Rhipicephalus microplus*- gut glycoprotein (Bm86) and *Ixodes scapularis-*Subolesin (4D8)	Bacterial (*Escherichia coli*)	**Dose:** 100 μg each antigen	*Rhipicephalus microplus*	Bm86: 79%	99% * Number of engorged ticks, oviposition and larval emergence	[97]
		**Adjuvant:** Montanide ISA50V2 (Seppic France).	*Rhipicephalus annulatus*	4D8: 58 ± 11% * Number of engorged ticks and oviposition		
		**Intervals:** four weeks (three doses)				
		**Model:** Cattle				
*Ixodes ricinus-*Recombinant gut glycoproteins: 86-1 (rIr86-1) and 86-2 (Ir86-2)	Bacterial (*Escherichia coli*)	**Dose:** 50 μg per antigen	*Ixodes ricinus*	rIr86-1 and Ir86-2	Overall efficacy data not reported.	[93]
		**Adjuvant:** Freundś complete adjuvant (FCA) (Priming dose) and Freundś incomplete adjuvant (FIA) (booster doses)		Overall efficacy data not reported.	* Number of engorged ticks and oviposition	
		**Intervals:** 3 weeks (3 doses)		* Number of engorged ticks and oviposition		
		**Model:** Rabbits				
*Rhipicephalus appendiculatus* histamine binding proteins (HBPM, HBPF1, HBPF2),	Bacterial (*Escherichia coli*)	**Dose:** 50 μg per antigen	*Rhipicephalus*	Not done	Overall efficacy data not reported.	[98]
*R. appendiculatus-*cement cone full-protein (TRPFL) and truncated-TRP protein (TRP18-89)		**Adjuvant:** Montanide ISA 50 V.	*appendiculatus*		* Number of engorged ticks and egg weight	
*R. appendiculatus-*subolesin homologue (4D8), and *Theileria parva* sporozoite antigen (p67C)		**Intervals:** four weeks (three doses)	*Theileria parva*			
		**Model:** Cattle				
*Rhipicephalus microplus-*protein: 39 (Rm39), 180 (Rm180), 239 (Rm239) and 76 (Rm76)	Bacterial (*Escherichia coli*)	**Dose:** 100 μg and 25 μg	*Rhipicephalus* (*Boophilus*) *microplus*	Note done	73.2% * Number of engorged ticks, oviposition and larval emergence	[117]
		**Adjuvant:** Aluminium hydroxide				
		**Intervals:** three weeks (three doses)				
		**Model:** Cattle				
*Amblyomma variegatum-*Recombinant Glutathione-S-transferase (rGST-Av) and *Rhipicephalus decoloratus-*Recombinant Glutathione-S-transferase (rGST-Rd)	Bacterial (*Escherichia coli*)	**Dose:** 100 μg per antigen	*Rhipicephalus sanguineus*	Note done	37.27% * Number of engorged ticks	[45]
		**Adjuvant:** Montanide 888 (Seppic) and Marcol 52.				
		**Intervals:** 14 days (three doses)				
*Rhipicephalus australis-*peptide: 4, 6 and 7 conjugated with key limpet haemocyanin (KLH)	Chemical synthesis	**Dose:** 200 μg	*Rhipicephalus australis*	Peptide 4-KLH 65%	47% * Number of engorged ticks, tick number/egg weights, and egg fertility	[72]
		**Adjuvants:** Freundś complete adjuvant (FCA) (Priming dose), Freundś incomplete adjuvant (FIA) (booster doses)		Peptide-6-KLH 63%		
(Peptide 4-KLH, Peptide-6-KLH, Peptide 7–KLH)		**Intervals:** Day 0, 4, 7 weeks (three doses)		Peptide 7–KLH 45%		
		**Model:** Cattle *Bos taurus* Herefords		* Number of ticks, tick number/egg weights, and egg fertility		
*Amblyomma variegatum-*Recombinant Subolesin (rSUB-Av),	Bacterial (*Escherichia coli*)	**Dose:** 100 μg	*Rhipicephalus appendiculatus*	rSUB-Ra: 47%, 50% (*B. indicus*) and 90%, 89% and 51% (cross-breed)	92%, 51% (*B. indicus*) and 74%, 69% and 71% (cross-breed)	[113]
*Rhipicephalus appendiculatus-*Recombinant Subolesin (rSUB-Ra)		**Adjuvant:** Montanide ISA50V2 (Seppic France)	*Rhipicephalus decoloratus*	rSUB-Ra: 68%, 58% (*B. indicus*) and 89%, 94% and 69% (cross-breed)		
		**Interval:** 30 days (two doses)	*Amblyomma variegatum*	rSUB-Av: 86%, 47% (*B. indicus*) and 83%, 76% and 72% (cross-breed)	* Number of engorged ticks, egg oviposition and egg fertility.	
*Rhipicephalus decoloratus-*Recombinant Subolesin (rSUB-Rd)		**Model:** Cattle (*Bovis indicus* and cross-breed)		* Number of engorged ticks, egg oviposition and egg fertility		
*Ornithodorus erraticus midgut-*epitope-based recombinant proteins: chitinases (OeCHl), 60S acidic ribosomal protein P0 (OeRPP0), secreted protein PK-4 (OePK4) and tetraspanins (TSPs = OeTSP1 + OeTSP2)	Bacterial (*Escherichia coli*)	**Dose:** 100 μg	*Ornithodorus erraticus* and	*O. erraticus:* OeCHl (30.2%), OeRPP0 (57.5%), OePK4 (57.8%) and (TSPs = OeTSP1 + OeTSP2) (56%)	*O. erraticus:* OeCHl + OeRPP0 + OePK4 + TSPs (66.3%)	[175]
		**Adjuvant:** Montanide ISA 50 V2 (Seppic, France)	*O. moubata*	*O. moubata:* OeCHl (19.6%), OeRPP0 (0%), OePK4 (8.1%) and (TSPs = OeTSP1 + OeTSP2) (11.1%)	*O. moubata:* OeCHl + OeRPP0 + OePK4 + TSPs (25.6%)	
		**Interval:** 14 days (three doses)		* Reduction of: ingested blood (in males, females and Nymph-3), mortality (of males, females and Nymph-3), moulting (of nymphs-3), oviposition (females) and fertility (females		
		**Model**: rabbits			* Reduction of ingested blood (in males, females, and Nymph-3), mortality (of males, females, and Nymph-3), moulting (of Nymph-3), oviposition (females) and fertility (females	
